# Optimization and validation of an ELISA assay for the determination of antibody responses to CN54gp140 and AIDSVAX BE for use in the Phase IIb PrEPVacc vaccine trial

**DOI:** 10.1371/journal.pone.0275927

**Published:** 2022-11-03

**Authors:** Ben Gombe, Claire Streatfield, Lorna Leal, Solomon Opio, Sarah Joseph, Jonathan Weber, Jonathan Hare, Pontiano Kaleebu, Jennifer Serwanga

**Affiliations:** 1 MRC/UVRI and LSHTM Uganda Research Unit, Entebbe, Uganda; 2 Uganda Virus Research Institute, Entebbe, Uganda; 3 IAVI Human Immunology Laboratory, Imperial College, London, United Kingdom; 4 AIDS and HIV Infection Research Group, IDIBAPS, Barcelona, Spain; 5 Faculty of Medicine, Imperial College, London, United Kingdom; 6 IAVI, New York, New York, United States of America; 7 London School of Hygiene and Tropical Medicine (LSHTM), London, United Kingdom; Consejo Nacional de Investigaciones Cientificas y Tecnicas, ARGENTINA

## Abstract

PrEPVacc is an international, multi-centre, double-blind vaccine study comparing experimental combination vaccine regimens including DNA/AIDSVAX BE and DNA/CN54gp140 with placebo control. Simultaneously, daily oral PrEP is compared for efficacy against daily Truvada in the context of the current PrEP availability situation at the study sites. An important clinical trial outcome is the accurate measurement of in vivo antibody titer induced through vaccination. Here we report the validation of two ELISAs for CN54gp140 and AIDSVAX BE at Uganda Virus Research Institute that demonstrates precision, specificity, and robustness for assessing the reciprocal antibody end point titer in human serum. This is a critical endpoint for determining whether vaccination can provide any protection against HIV in populations at risk of acquiring HIV.

## Introduction

Despite the widespread rollout of antiretroviral therapy (ART), HIV remains a major global health challenge and continues to disproportionately affect those living in Sub-Saharan Africa with 20.6 million people living with the virus and 670,000 new infections in 2020 and accounting for approximately 54% of the total worldwide [1]. Vaccination is still considered the most cost-effective strategy for the control and eradication of infectious diseases but despite over 30 years of sustained research and 6 vaccine efficacy trials, there is still no HIV vaccine [2].

It has still not yet proved possible to elicit broadly neutralizing antibodies (bnAbs) with a vaccine but the identification, characterization and production of bNAbs with increased breadth and potency has provided an opportunity to explore the potential of passively administered antibodies to reduce viremia or delay viral rebound following ART interruption in HIV-infected individuals [3–6]. The ability of bnAbs to protect at a population level was assessed in the Antibody Mediated Protection studies (AMP) and found to have measurable effectiveness at preventing acquisition of neutralization susceptible circulating strains of HIV. Induction of Env-specific non-neutralizing antibodies through vaccination have been more successful, with the results of the RV144 trial being the first HIV vaccine trial to report efficacy [7]. Post hoc correlates analysis found a lower risk of HIV-1 infection in vaccine recipients whose plasma IgG bound an antigen comprising the gp120 variable regions 1 and 2 (V1V2) of Env attached to the C terminus of a murine leukaemia virus (MLV) gp70 scaffold (gp70-V1V2) [8], [9]. Subsequent studies, including sequence analysis of “breakthrough viruses” in infected individuals and functional assessment of isolated monoclonal Abs, further strengthened the case for a role for non-neutralising antibodies in protection and provided a plausible mechanism for their action through antibody dependent cell-mediated cytotoxicity [10]. More recent data has emerged from HVTN702 that indicates that a vaccination strategy based primarily on the induction of non-neutralising antibodies was unable to recapitulate the protection observed in RV144. Whilst the analysis is ongoing to untangle explanations for these discordant data sets, further studies that add to this data set are important for improving our understanding of the contribution that non-neutralizing antibodies play in mediating protection from HIV-1 infection.

The use of daily antiretrovirals (ARV) has been much more successful as a prophylactic intervention and has been recommended, among other prevention approaches, since 2015 by the WHO for populations with an HIV incidence above 3% [11]. Further advances have led to the development of long-acting antiretrovirals such as the cabotegravir and rilpivirine combination which has recently received regulatory approval from FDA, EMA and Health Canada. Long-acting ARVs aim to decrease the 365 daily pill burden to only six intramuscular injections per year with hopes for further advancements towards potential formulations that can be administered as an implant with a dosing interval of 1 year or more [11].

PrEPVacc is an Africa-led Phase IIb trial assessing the safety and efficacy of two different vaccination regimens given with pre-exposure prophylaxis regimens in which a new form of oral PrEP (Descovy) taken daily is compared for efficacy against daily Truvada in the context of the current PrEP availability situation at the study sites (www.prevacc.org). The selected antigens for PrEPVacc, AIDSVAX BE and CN54gp140, have been tested extensively in previous clinical trials with binding antibody titers determined by antigen-specific ELISA [7, 12, 13]. In this study we describe the development of a harmonized enzyme-linked immunosorbent assay (ELISA) protocol for determining the titer of human IgG binding antibodies directed against CN54gp140 and AIDSVAX BE recombinant proteins. The two ELISAs have been validated at Uganda Virus Research Institute (UVRI) according to relevant guidelines [14] with demonstrated precision, specificity, and robustness in preparation for receiving and testing clinical samples as the primary immunogenicity assay readout for PrEPVacc.

## Materials and methods

### Coating antigens

CN54 is a recombinant HIV gp140 envelope protein produced by Polymun Scientific, Klosterneuberg, Austria. The protein was stored in aliquots at -20°C until use.

AIDSVAX BE is a combination product comprising two HIV gp120 envelope recombinant proteins (MN and A244). The protein was stored in aliquots at -80°C until use. The constituent proteins were mixed prior to use in accordance with instructions provided by the manufacturer (VaxGen).

### Test serum samples and controls

The assay was optimised using test serum samples from vaccinated volunteers within the HIV clinical trials Tamovac II (NCT01697007) and IDEA EV06 (NCT02376582). These samples had previously well characterised responses to AIDSVAX BE and CN54gp140 proteins and included negative (baseline) and positive responses of varying magnitude. Negative control serum samples were prepared as a pool derived from healthy, unvaccinated adults living in regionally relevant areas as well as 21 commercially procured serum samples. As no international standard preparation is available, a positive control sample for was prepared as a serum sample pool derived from individuals previously vaccinated with AIDSVAX or CN54gp140. All study participants and serum donors had given written informed consent for the use of their serum for vaccine research.

To allow tracking and trending of assays over time, a high and low QC sample were prepared for both AIDSVAX BE and CN54 gp140 methodologies. These samples were derived from the positive control samples used for assay precision in which a dilution was selected to return a fixed and reproducible absorbance value of ~1.0 and ~0.3, respectively (S1 Table in S2 Data).

The application of the test serum samples and controls during the validation is outlined in Table 1.

**Table 1:** Validation assay parameters and control samples.

Parameter	Definition	Reagent	Sample descriptions
Precision	Defined as the closeness of agreement between independent test results obtained under stipulated conditions	Positive Control	2 pools of samples for use in titration curves
Specificity	Determines the capability of the method to assess the analyte in the presence of other components	Test Samples	94 human serum samples from HIV-uninfected individuals sampled from USA, Tanzania and Uganda
Robustness	Robustness is a measure of the assay’s capacity to remain unaffected by small but deliberate variations in method parameters, which indicated its reliability under anticipated conditions	Positive Control	2 pooled samples for use in titration curves
LOD	Lowest concentration of an analyte that can be detected but not necessarily quantified	NA	NA
Assay Performance	To ascertain that the quality does not degrade over time and provide criteria to determine if a run is accepted.	QC Samples	4 QC samples for assay tracking (2 high and 2 low). 1 Negative serum pool

### CN54 ELISA methodology

The ELISA to measure CN54gp140 was based on previously published methods [13]. Briefly, CN54gp140 protein was coated (50 μL/well of a solution of 1.0 μg/mL prepared in Dulbecco’s Phosphate Buffered Saline (PBS)) onto a medium binding, flat-bottomed 96-well polystyrene plate (Greiner Bio-One) overnight (16 hours) at 2–8°C.

Plates were washed manually four times (250 μL/well of PBS supplemented with 0.05% Tween-20 (PBST)), and gently blotted on clean blotting paper. Plates were then blocked with assay buffer (200 μL/well PBST supplemented with 1% Bovine Serum Albumin (1% BSA/PBST)) to saturate nonspecific binding sites, for 1 hour at 37°C. Plates were then washed four times with 0.05% PBST and gently blotted dry.

All sample dilutions and controls were prepared in tissue-culture treated polystyrene, U-bottom plates using assay buffer (1% BSA/PBST). Sample dilutions for specificity testing were prepared at 1:100 dilutions. Standard titration curves were prepared as an 11-point dilution curve with 2-fold dilutions. QC standards were prepared as ready to use aliquots. All samples were plated in triplicate.

Samples and controls, blank, were added (50 μL/well) and plates were incubated for 1 hour at 37°C. Plates were washed four times with 0.05% PBST and gently blotted before adding horseradish peroxidase-conjugated anti-human IgG goat antibody (Sigma, A0170) diluted 1:10000 in assay buffer (1% BSA/PBST) for 1 hour at 37°C. After washing plates four times with 0.05% PBST and gently blotting, the substrate was added (50 μL/well of Sureblue™ 3,3’,5,5’-Tetramethylbenzidine (TMB) 1-Component Peroxidase Substrate). The TMB is oxidized by HRP using hydrogen peroxide, which will result in a blue coloured product. This reaction was then terminated, after 5 minutes protected from direct light, by the addition of a HCL-based stop solution (50 μL/well of TMB Stop Solution), which changed the color change from blue to yellow, due to pH changes. The optical density (OD) was determined by reading the assay plate at a wavelength of 450nm using a Biotek 800TS reader and Gen5 software and is directly correlated to the amount of target specific antibody binding in the serum.

### AIDSVAX BE ELISA methodology

The AIDSVAX BE ELISA methodology was performed as previously described by Pitisuttithm et al [12]. Briefly, AIDSVAX BE protein was prepared by combining equal amounts of MN rgp120 and A244 rgp120 in Dulbecco’s Phosphate Buffered Saline (Sigma D8537) at a final concentration of 2.0μg/mL. 50μL/well of combined protein solution was transferred to each well of a medium binding, flat-bottomed 96-well polystyrene plate (Greiner Bio-One) overnight (16 hours) at 2–8°C.

Plates were washed manually four times (250 μL/well of PBS supplemented with 0.05% Tween-20 (PBST)), and gently blotted on clean blotting paper. Plates were then blocked with assay buffer (200 μL/well PBST supplemented with 1% Bovine Serum Albumin (1% BSA/PBST)) to saturate nonspecific binding sites, for 1 hour at 37°C. Plates were then washed four times with 0.05% PBST and gently blotted dry.

All sample dilutions and standards were prepared in tissue-culture treated polystyrene, U-bottom plates using assay buffer (1% BSA/PBST). Sample dilutions for specificity testing were prepared at 1:100 dilutions. Standard titration curves were prepared as an 11-point dilution curve with 2-fold dilutions. QC standards were prepared as ready to use aliquots. All samples were plated in triplicate.

Samples and controls, blank, were added (50 μL/well) and plates were incubated for 1 hour at 37°C. Plates were washed four times with 0.05% PBST and gently blotted before adding horseradish peroxidase-conjugated anti-human IgG goat antibody (Sigma, A0170) diluted 1:10000 in assay buffer (1% BSA/PBST) for 1 hour at 37°C. After washing plates four times with 0.05% PBST and gently blotting, the substrate was added (50 μL/well of Sureblue™ 3,3’,5,5’-Tetramethylbenzidine (TMB) 1-Component Peroxidase Substrate). The TMB is oxidized by HRP using hydrogen peroxide, which will result in a blue coloured product. This reaction was then terminated, after 5 minutes protected from direct light, by the addition of a HCL-based stop solution (50 μL/well of TMB Stop Solution), which changed the color change from blue to yellow, due to pH changes. The optical density (OD) was determined by reading the assay plate at a wavelength of 450nm using a Biotek 800TS reader and Gen5 software and is directly correlated to the amount of target specific antibody binding in the serum.

#### Precision

Precision is defined as the closeness of agreement between independent test results obtained under stipulated conditions, called intra assay, inter assay and inter operator precision. To determine precision, a serum pool from vaccinated individuals was prepared spanning a dilution range of 1:100–1:102400. For intra assay precision, similar samples were measured at the same occasion in triplicate by a single operator on five separate plates; for inter assay precision, samples were measured in triplicate by a single operator on 5 separate occasions and for inter operator precision, samples were measured in triplicate by two operators on 5 separate occasions.

#### Specificity

The specificity of an analytical method is used to determine the capability of the method to assess the analyte in the presence of other components, such as are impurities, degradation products and/or sample matrix which may have an impact. Assay specificity was defined as the potential of the test to correctly assess the proportion of true test-negatives. It was used as a measurement of how well the assay performed in a group of known HIV-1 negative and non-vaccinated individuals. Specificity was assessed by testing 94 serum samples from individuals confirmed as HIV negative. The samples were assessed in triplicate at a 1:100 fixed dilution.

#### Robustness

Robustness is a measure of the assay’s capacity to remain unaffected by small but deliberate variations in method parameters, which indicated its reliability under anticipated conditions. Robustness evaluation was performed in two phases. Phase one evaluated manual plate washing against using an automated plate washer and then comparing readouts between two microplate readers BioTek 800TS and Tecan Infinite M200 Pro). Phase two evaluated varying the coating incubation times (16h, 40h, 64h, 88h, 112h, 136, 160h), plate blocking times (55, 60 and 65 minutes), sample incubation times (55, 60 and 65 minutes) and detection antibody incubation times (55, 60 and 65 minutes). These are the key steps within the methodology at which variability can be introduced.

#### Determination of assay characteristics

The lower limit of detection (LLOD) is the lowest concentration of an analyte that can be detected but not necessarily quantified. The assay limit of detection (LOD) was evaluated by fitting four parameters logistic (4-PL) transformation to the data derived from Inter operator precision and determining the 99% confidence interval (99% CI) from the lower asymptote. For a standard curve, the LOD was the concentration corresponding to the interpolated intersection of the upper 99% CI of the lower asymptote with the 4-PL curve fit.

The statistical cut-off of the ELISA was based on the upper limit of the 99.9% one-sided confidence interval of the specificity testing in HIV negative samples. The optical density (OD) corresponding to the dilution of each sample were averaged and the standard deviation was determined.

All assays had to meet assay acceptance criteria to be considered a valid result (S2 Table in S2 Data).

### Statistical analysis

Optical density absorbance values at 450nm were exported from BioTek Gen5 software for further analysis. Statistical analyses were performed on log10-transformed data using GraphPad Prism v9.3. For precision, computations were performed on the transformed values but the variability in terms of CV was expressed relative to the non-transformed dilutions. Correlation analysis for harmonization, precision was determined using Pearson correlations. Sensitivity analysis used untransformed data and analysis utilized Spearman Rank correlation.

To evaluate the effect of comparing different conditions and equipment, each individual factor was considered as fixed and the variance to that was measured. Evaluation of plate washing (manual vs automated) and plate readers (BioTek vs Tecan) was compared by means of a paired t-test with incubation parameter testing assessments using a one-sided Dunnett test [15].

The quality control (QC)-specifications of each of the control samples were defined by computing standard deviation through all data. This data was plotted using Levey-Jennings plots to measure assay performance following the Westgard rules [16, 17]. Additional analysis of QC sample data used t-test and Welch’s t-test [18]. P values ≤0.05 were considered significant.

## Results

### Method harmonization

**Fig 1:**
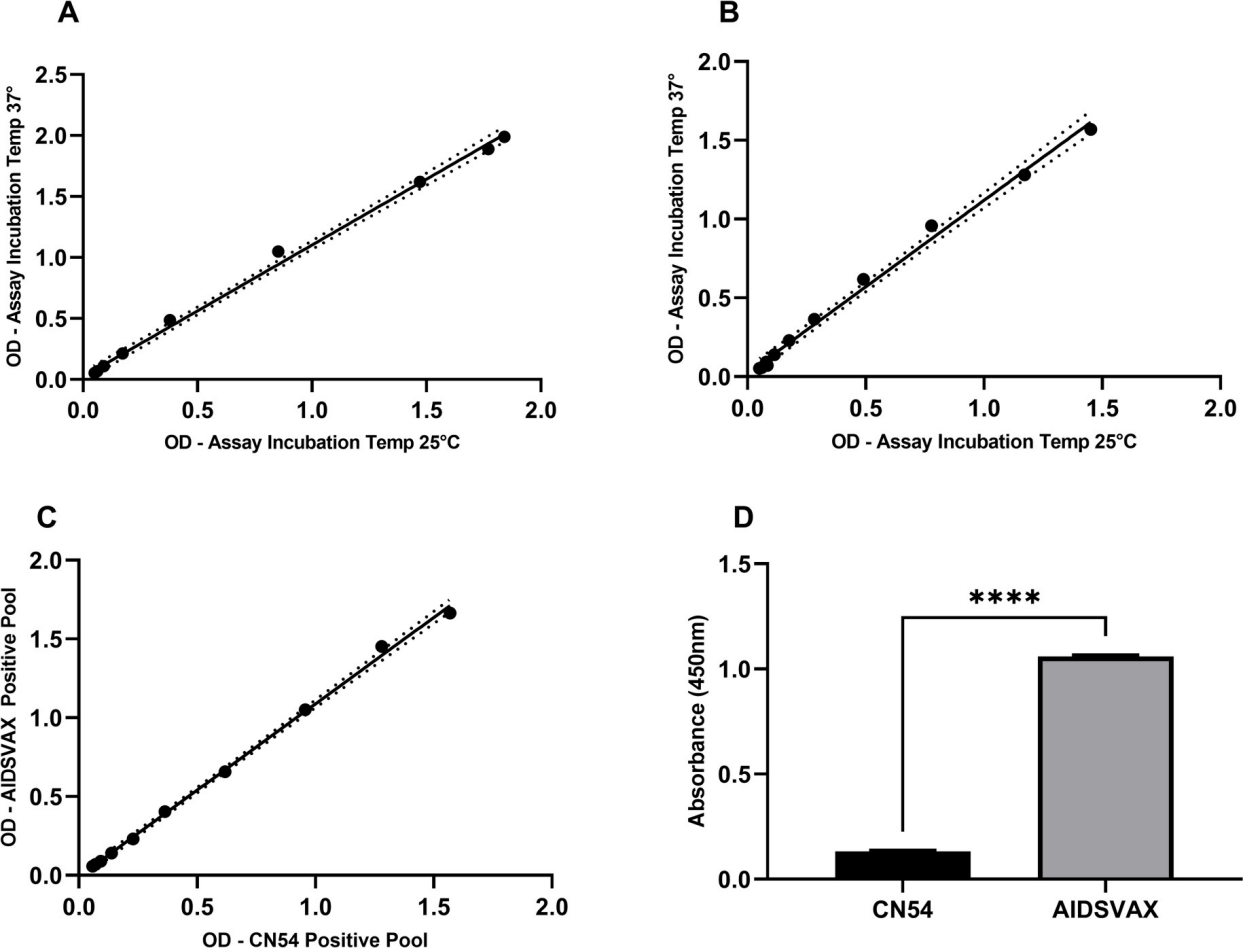
Method harmonization. A–Comparison of assay incubation temperature (25°C vs 37°C) for AIDSVAX BE methodology. B–Comparison of assay incubation temperature (25°C vs 37°C) for CN54 methodology. C–Comparison of AIDSVAX BE and CN54 positive serum pool. D–Comparison of AIDSVAX BE High QC sample evaluated on the CN54 ELISA and AIDSVAX BE ELISA.

To enable a single methodology to be developed for assessing responses to both CN54 and AIDSVAX BE the key steps identified for harmonization were assay incubation temperature, the preparation of QC samples and serum pools for titration curves. Fig 1 shows that performing both the AIDSVAX BE (Fig 1A; r = 0.9985, p<0.0001) and CN54 (Fig 1B; r = 0.9970, p<0.0001) ELISA methodology at either 37°C or 25°C had no impact on assay performance. QC samples were prepared by pooling vaccine sero-positive serum samples from confirmed positive samples for CN54 and AIDSVAX BE antibodies. A comparison of serial dilutions for serum pools showed good concordance for both CN54 and AIDSVAX BE (Fig 1C). However, differences were pronounced for the AIDSVAX BE High QC sample with significantly lower absorbance observed when assessed against CN54 as a capture antigen (Fig 1D p<0.0001), as such separate QC samples were then prepared for use in the CN54 and AIDSVAX BE ELISA respectively (see S1 and S2 Figs in S2 Data for assessment of vaccine matched seropositve samples and QC sample data).

### Assay validation

#### Precision

**Fig 2:**
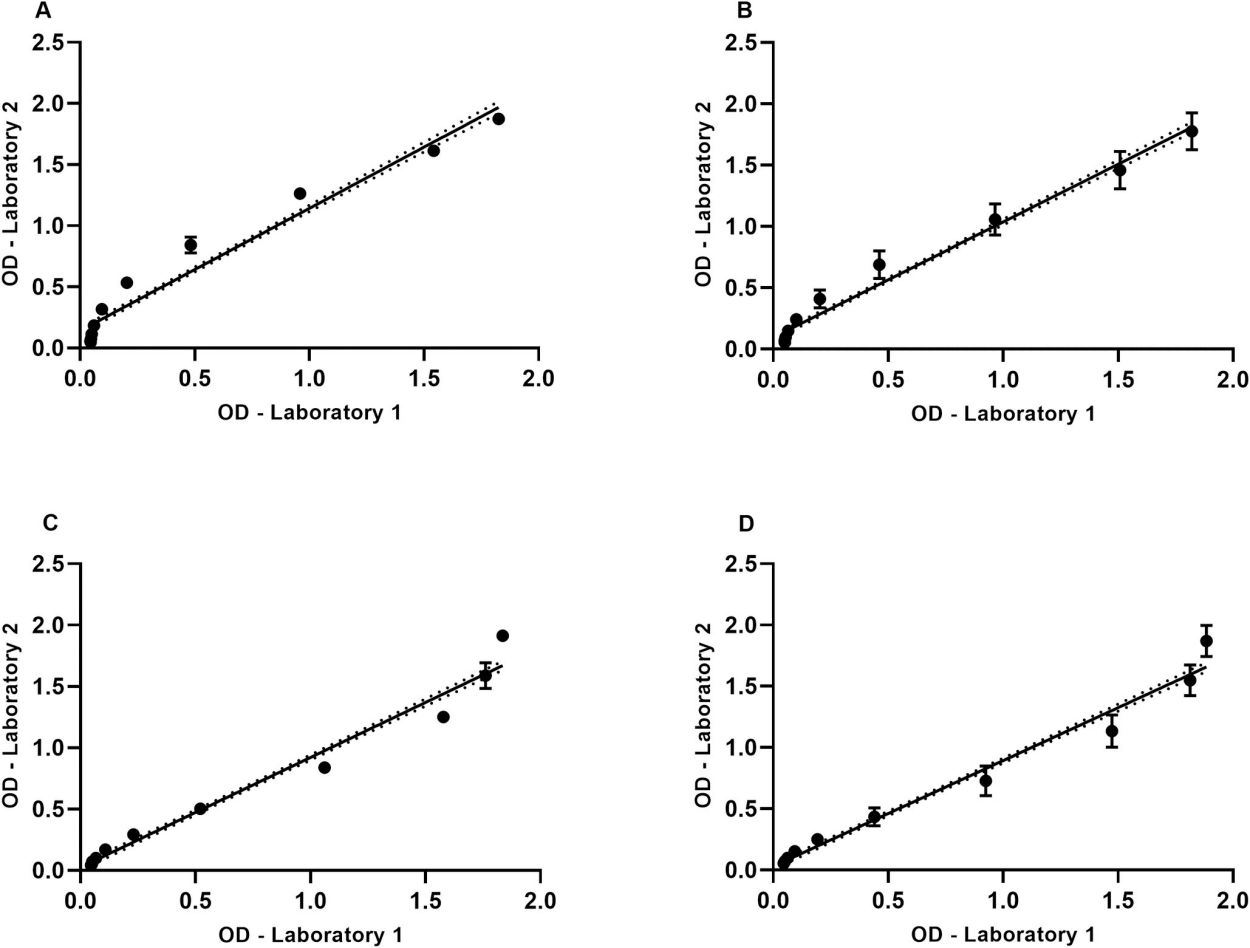
Precision assessment comparing values obtained through optimization phase and validation phase. A–CN54 Intra Assay correlation p<0.0001. B–AIDSVAX BE Intra Assay correlation p<0.0001. C–CN54 Inter Operator correlation p<0.0001. B–AIDSVAX BE Inter Operator correlation p<0.0001.

Precision was considered at two levels: repeatability (intra assay and inter assay) and intermediate (inter operator). To determine intermediate precision, positive serum pools derived from vaccinated subjects were titrated and tested by different operators on different days at two laboratories. Fig 2 shows a correlation for intra assay and intermediate precision between the two laboratories (UVRI and IAVI Human Immunology Laboratory)

Assay precision was determined using full data sets derived from Laboratory 2 (UVRI). The %CV for intra assay, inter assay and intermediate precision are shown in Table 2. Examination of the data showed low variability for Intra assay precision with an average variability of 6.2% and 6.3% for CN54 and AIDSVAX BE respectively (range: 2.45–12.56%); 13.0% and 10.7% for inter assay precision (range: 6.24–23.84%) and an average variability 12.3% and 12.1% (range: 6.80–18.04%) for intermediate precision. There was a trend for increased variability as the serum dilution series increase, with a maximum variability of 24% observed for AIDSVAX BE inter assay precision at positive serum dilution of 1:25600. This precision data was in line with the requirements as defined in the Material and Methods section (for full precision values see S3-S5 Tables in S2 Data).

**Table 2:** Summary of %CV assessment determined through precision assessment for CN54 and AIDSVAX BE ELISA methodology. No. Values = the number of datapoints use for calculating precision.

Serum Titration	Intra Assay Precision				Inter Assay Precision				Intermediate Assay Precision			
	No. Values		%CV		No. Values		%CV		No. Values		%CV	
	CN54	AVAX	CN54	AVAX	CN54	AVAX	CN54	AVAX	CN54	AVAX	CN54	AVAX
1:100	15	12	2.47	2.45	15	15	8.66	8.84	15	15	8.51	6.80
1:200	15	12	2.90	6.63	15	15	10.63	7.59	15	15	10.42	7.98
1:400	15	12	3.87	3.01	15	15	14.61	8.52	15	15	11.95	11.49
1:800	15	12	7.72	4.10	15	15	18.53	8.77	15	15	16.40	16.62
1:1600	15	12	4.75	5.41	15	15	18.95	10.43	15	15	18.04	17.07
1:3200	15	12	5.71	3.60	15	15	18.47	8.32	15	15	15.59	17.98
1:6400	15	12	4.94	5.21	15	15	16.17	7.58	15	15	14.04	11.74
1:12800	15	12	5.74	6.33	15	15	14.31	6.24	15	15	8.91	11.00
1:25600	15	12	11.87	8.56	15	15	7.56	23.84	15	15	11.40	12.34
1:51200	15	12	9.56	11.09	15	15	7.97	22.15	15	15	8.56	8.95
1:102400	15	12	8.29	12.56	15	15	7.33	5.79	15	15	11.38	10.81

#### Specificity and cut-off determination

To assess non-specific background activity in human serum, 94 HIV-1 seronegative sera (sampled from USA, Tanzania and Uganda) were diluted at 1:100 and tested for non-specific background binding. Absorbance values (excluding samples that did not meet QC acceptance criteria) were used to calculate an ELISA specific positivity threshold to allow the distinction between CN54 and AIDSVAX BE negative and positive samples.

**Fig 3:**
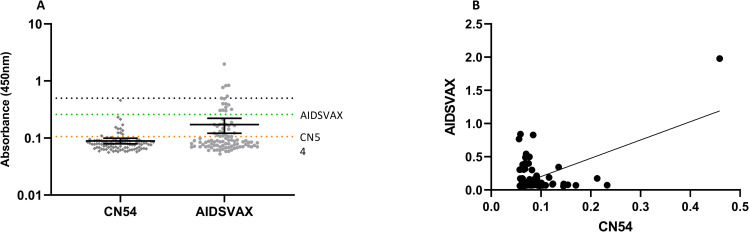
Assay Cut-off determination for CN54 and AIDSVAX BE ELISA. A–Assay cut-off determination. Orange line represent calculated assay cut off for CN54 ELISA. Green dotted line represents calculated assay cut off for AIDSVAX BE ELISA. Grey dotted line represents final assigned assay cut-off. B–CN54 and AIDSVAX BE sensitivity sample correlation analysis.

Specificity evaluation showed an elevated background absorbance for AIDSVAX BE compared to CN54 and this translated to elevated assay cut-off using calculation (0.259 compared to 0.106 –Fig 3A). Correlation of the mean absorbance values indicated a positive association between the two methodologies (Fig 3B. r = 0.544, p = <0.0001). Whilst the correlation was limited, the data indicated general positivity of samples for both CN54 and ADSVAX BE with a tendency to elevated reactivity to AIDSVAX BE compared to CN54.

Using the prescribed assay cut-off values would translate to an observed false-positive rate of 12.7% and 16.0% for CN54 and AIDSVAX BE ELISA respectively. The final cut-off used for clinical immunogenicity evaluation will be fixed at an absorbance value of 0.200 and 0.500 for the CN54 and AIDSVAX BE ELISA methodologies respectively. This would translate to an expected false positive rate and observed false-positive rate of 0 and 5.3% for CN54 and AIDSVAX BE ELISA respectively.

#### Lower Limit of Detection (LLOD)

**Fig 4:**
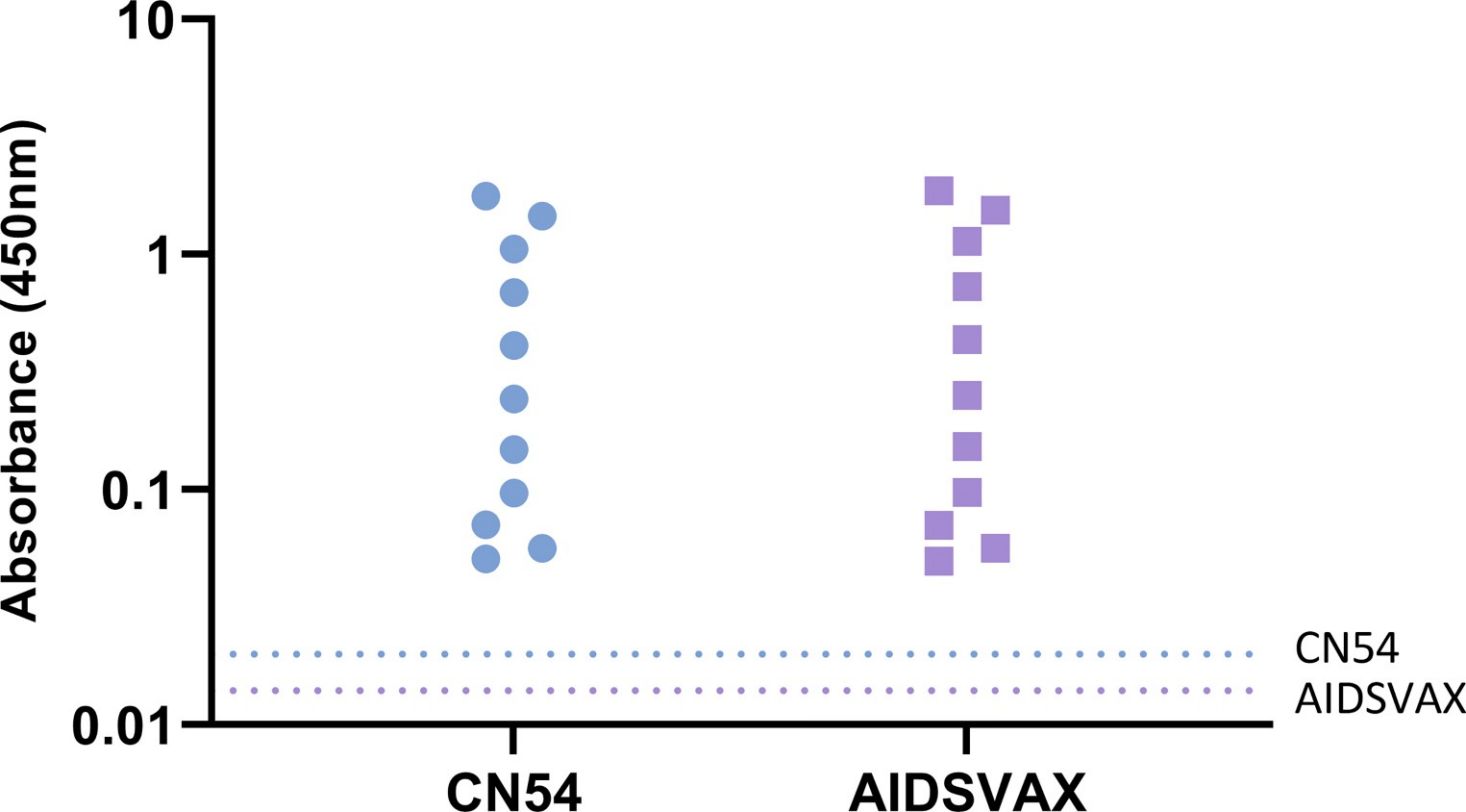
Limit of detection determination. Blue dotted line– 99% CI from Lower asymptote (CN54 ELISA). Purple dotted line– 99% CI from Lower asymptote (AIDSVAX BE ELISA).

The lower limit of detection (LLOD) was determined by fitting four parameters logistic (4-PL) transformation to the data derived from Inter Operator Precision and determining the 99% confidence interval (99% CI) from the lower asymptote. This calculation recorded a value is 0.020 for CN54 ELISA and 0.014 for AIDSVAX BE ELISA (Fig 4).

#### Robustness

Six different parameters were assessed during robustness testing for each assay, split in to two phases. Phase 1 compared manual washing steps with the use of an automated plates washer and evaluated determining absorbance on two independent plate readers. Paired student t-tests were performed on the data sets along with an evaluation of the IC50 values for the different conditions. Comparison of manual and automated plate washing showed no difference between manual and automated plate washers (p>0.05, Table 3; S3A-S3D Fig in S2 Data).

**Table 3:** Statistical comparison of robustness phase 1 parameters using paired student’s t-test.

CONDITION	CN54	AIDSVAX BE
*Plate washing* manual vs automated	0.057	0.161
*Plate readers* Tecan Infinite vs Biotek	0.154	0.300

Phase 2 of robustness testing compared varying the incubation time for antigen coating (16–168 hours), varying the plate blocking times (60 minutes ±5 minutes), varying the sample incubation times (60 minutes ±5 minutes) and varying the detection antibody times (60 minutes ±5 minutes). No differences were observed for any of the conditions tested (Table 4 and S3E-S3L Fig in S2 Data).

**Table 4:** Statistical comparison of robustness phase 2 parameters. Data shown are Dunnett’s test p values.

CONDITION	CN54	AIDSVAX BE
*Antigen coating* 16hrs vs 40hrs	1.000	0.999
*Antigen coating* 16hrs vs 64hrs	1.000	0.999
*Antigen coating* 16hrs vs 88hrs	0.999	1.000
*Antigen coating* 16hrs vs 112hrs	0.999	1.000
*Antigen coating* 16hrs vs 136hrs	0.999	1.000
*Antigen coating* 16hrs vs 136hrs	1.000	0.999
*Plate blocking* 60 mins vs 55mins	0.989	0.999
*Plate blocking* 60 mins vs 65mins	0.982	0.999
Sample incubation 60 mins vs 55mins	0.998	0.992
*Sample incubation* 60 mins vs 65mins	1.000	0.997
*Detection antibody incubation* 60 mins vs 55mins	0.999	1.000
*Detection antibody incubation* 60 mins vs 65mins	0.999	0.998

#### QC samples

**Fig 5:**
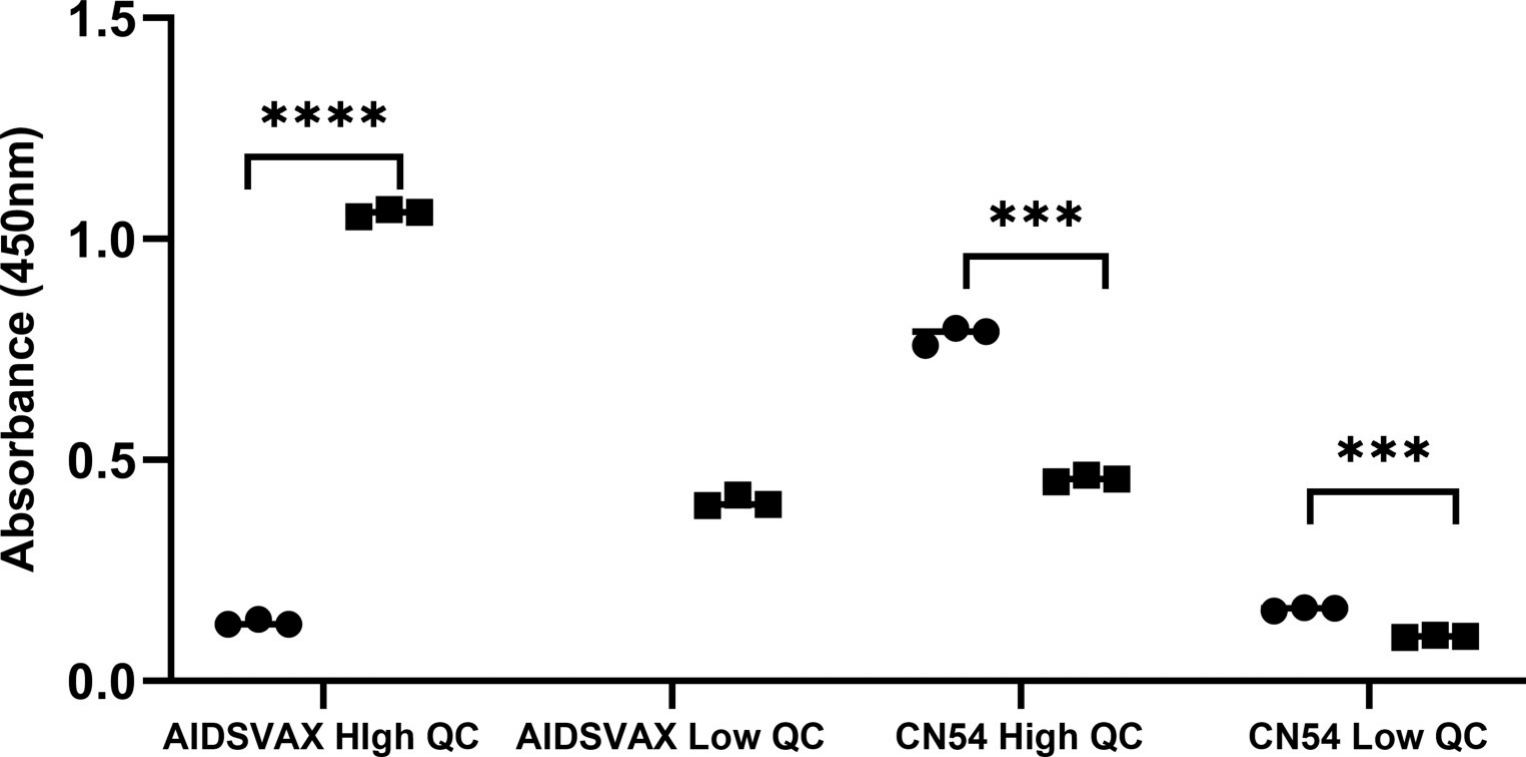
QC samples comparison. Comparison of QC control sample performance using CN54 and AIDSVAX BE ELISA methodology. Circles–CN54 ELISA, Squares–AIDSVAX BE ELISA. Statistical analysis using student’s t-test.

The consistency of assay readouts were evaluated using a serum pool prepared from serum samples from volunteers who had received either CN54 or AIDSVAX BE vaccines within the EV06 and Tamovac II trials. Comparison of the samples indicated that although there was some cross reactivity between the CN54 High QC positive sample tested with the AIDSVAX BE ELISA there was no cross reactivity of AIDSVAX BE positive samples with the CN54 ELISA (Fig 5). Notwithstanding the cross-reactivity observed there was an observable significant difference in signal sensitivity when using matched samples to capture antigen.

Due to the observed cross-reactivity between the serum samples it was necessary to have two independent QC sample sets for the CN54 and AIDSVAX BE ELISA. These QC samples were used to track assay consistency over time and were interpreted using Levey-Jennings plots and Westgard rules. Evaluation of the data obtained during assay optimization and validation did not show any breaches of the Westgard rules [17, 19] that would require an assay run to be invalidated, indicating that the assay performance was consistent over time (See S4 Fig in S2 Data).

## Discussion

ELISA has been the preferred methodology for measuring the levels of binding antibody responses within many of the late-stage HIV efficacy studies to date including RV144 [7] and HIVIS03 [20] that utilize the same protein vaccines as part of the ongoing PrEPVacc Phase IIb prophylactic HIV vaccine trial. The primary immunogenicity endpoint for PrEPVacc is the determination of end point antibody titers will be a secondary immunological outcome and will be determined using a standard ELISA methodology. The publication describes the validation of this ELISA in preparation for receiving and testing clinical samples at the MRC/UVRI and LSHTM Uganda Research Unit in Entebbe, Uganda as the central testing laboratory.

The results of the validation demonstrated that the harmonized ELISA methodology is specific, precise, and capable for repeatable titration of antibodies specific for CN54gp140 and AIDSVAX BE HIV envelope proteins with average precision values ranging from 6.2% to 13%. These values are consistent with established criteria for ELISA assays within the field, although there are no values specified within official guidance.

Determination of the assay-cut off was a key objective for the validation as this is the threshold for determining positivity in a clinical sample. This value was calculated by assessed by screening normal human serum samples from USA, Tanzania and Uganda and resulted in the determination of statistical cut-off of 0.106 and 0.259 for CN54 and AIDSVAX BE respectively. To facilitate a more stringent positivity threshold, the final cut-off used for clinical immunogenicity evaluation will be fixed at an absorbance value of 0.200 and 0.500 was applied for the CN54 and AIDSVAX BE ELISA methodologies.

Assessment of the sample origins revealed that there was no difference in values for all samples of different origins when tested using AIDSVAX BE methodology (S5 Fig in S2 Data). This was also the case using the CN54methodolofy comparing samples from Uganda and Tanzania. Differences were observed between USA derived samples and Tanzanian and Ugandan samples for CN54 but as these were consistently lower than these observed for African derived samples, they were included in cut-off determinations as any influence would be on the false positive rate, which has been mitigated through the selection of the elevated threshold.

Comparison of the cross-reactivity of Tamovac II and EV06 samples revealed limited cross reactivity when samples were assessed by matched vaccine ELISA and mis-matched ELISA. Broadly there was increased cross reactivity for AIDSVAX BE vaccinated samples assess using the CN54 ELISA than the reverse. This asymmetrical cross-reactivity between the two methodologies would account for the weak correlation when comparing sample sensitivity.

In completing this activity, we believe this is the first example of the primary immunogenicity end-point assay validation being led on-site at the primary immunology testing lab in sub-Saharan Africa, and as such is an important advancement in ensuring the sustainability of performing complex end point efficacy studies within the communities most affected.

### Limitations and recommendations

Assay validation followed the ICHQ2 guidance on validation of analytical procedures. The guidance does not specific fully how to perform any specific assay only that “experimental work can be designed so that the appropriate validation tests can be performed to provide sound, overall knowledge of the performance of the analytical procedure, for instance: specificity/selectivity, accuracy, and precision over the reportable range” [14]. The details of how each parameter is assessed is specific for each assay platform and objective and as such the validation of each assay will be unique to the desired outcome and available reagents. The guidance for precision outlines objectives assessing repeatability, intermediate precision, and reproducibility. As the assay will only be run at single lab reproducibility was not assessed. The guidance specifies that the minimum number of data points required be “either 9 determinations (3 replicates/3 conditions) covering reportable range or 6 at 100%” although the consistency in the literature is to increase the recommended range to 5 dilution points [21]. There is no guidance on the sample type for which to assess precision, however to cover the low availability of positive samples the use of a sample pool is widely accepted and has been demonstrated previously with examples available of the use of single pool [22]. The use of a pooled sample for standardisation has also been endorsed from the WHO where their approved standard for anti-Lassa fever virus antibodies is derived from “pooled human plasma” [23].

The use of two independent protein vaccines encoding different iterations of HIV envelope requires a twin ELISA system using both CN54 and AIDSVAX BE proteins as the capture antigens, doubling the effort for testing clinical samples. Coupled with the extensive effort in performing these assays is the observed assay sensitivity for ELISA assays. Whilst clinical samples are expected to have robust antibody responses induced through vaccination as observed during original clinical evaluations [7], [13], sensitivity of ELISA assays is typically 10-fold higher than either electrochemiluminescent or binding antibody multiplex assays [24]. Whilst development of a multiplex binding assay for both CN54 and AIDSAVX would address both limitations of the current strategy there would be significant additional challenges in setting up this platform and transitioning to these more advanced techniques should be considered for future studies.

## Supporting information

S1 Data (XLSX)

S2 Data (DOCX)
