# Supplementary material for: Optimization and validation of an ELISA assay for the determination of antibody responses to CN54gp140 and AIDSVAX BE for use in the Phase IIb PrEPVacc vaccine trial
Source: PLoS One. 2022 Nov 3;17(11):e0275927. doi: 10.1371/journal.pone.0275927 (PMC9632806; doi:10.1371/journal.pone.0275927)
Supplement: S2 Data — (DOCX) [file pone.0275927.s002.docx]

*S1 Figure. Comparison of binding antibody responses using human serum samples immunized with AIDSVAX (EV06) or CN54 (Tamovac II). A: CN54 Coating Protein. B: ADSVAX Coating Protein. Statistics – Welch’s t-test p<0.0001*

*S2 Figure. Comparison of QC samples. A: CN54 Coating Protein. B: AIDSVAX Coating Protein.*

*S3 Figure. A & B - Plate washing comparison. C & D – Plate reader comparison. E & F – Coating antigen incubation conditions. G & H – Plate blocking conditions. I & J – Sample incubation conditions. H & K – Detection antibody incubation conditions*

**

*S4 Figure. Levey-Jennings track and trend plotting of QC and negative samples for CN54 and AIDSVAX ELISA. Solid line – mean absorbance value, dashed line ±1 SD, dotted line ±2 SD, dashed and dotted line ±3 SD*

**

*S5 Figure. Comparison of sensitivity data. A – Samples from USA, Tanzania and Uganda tested using CN54 methodology. B – Samples from USA, Tanzania and Uganda tested using AIDSVAX methodology. Statistical assessment Mann-Whitney test. NS = P value >0.05. **** = p<0.0001.*

|  | *CN54* | | *AIDSVAX BE* | |
| --- | --- | --- | --- | --- |
| *Dilution 1* | *High QC* | *1:800* | *High QC* | *1:400* |
| *Dilution 2* | *Low QC* | *1:3200* | *Low QC* | *1:1600* |

*S1 Table. QC Sample Dilutions*

|  | *CN54* | *AIDSVAX BE* |
| --- | --- | --- |
| *High QC* | *>0.652* | *>0.766* |
| *Low QC* | *>0.088* | *>0.302* |
| *Negative Control* | *<0.079* | *<0.199* |

*S2 Table. QC Sample and control acceptance criteria*

| *Dilution Series* | *CN54 ELISA* | | | | | | *AIDSVAX ELISA* | | | | | |
| --- | --- | --- | --- | --- | --- | --- | --- | --- | --- | --- | --- | --- |
|  | Count | Min | Max | Average | STDEV | %CV | Count | Min | Max | Average | STDEV | %CV |
| 100 | 15 | 1.793 | 1.970 | 1.874 | 0.046 | 2.5 | 15 | 1.661 | 1.971 | 1.882 | 0.092 | 4.9 |
| 200 | 15 | 1.533 | 1.688 | 1.614 | 0.047 | 2.9 | 15 | 1.231 | 1.733 | 1.545 | 0.139 | 9.0 |
| 400 | 15 | 1.168 | 1.370 | 1.263 | 0.049 | 3.9 | 15 | 0.814 | 1.304 | 1.172 | 0.168 | 14.3 |
| 800 | 15 | 0.632 | 0.918 | 0.842 | 0.065 | 7.7 | 15 | 0.557 | 0.912 | 0.796 | 0.099 | 12.4 |
| 1600 | 15 | 0.49 | 0.586 | 0.535 | 0.025 | 4.8 | 15 | 0.286 | 0.556 | 0.467 | 0.079 | 16.9 |
| 3200 | 15 | 0.281 | 0.355 | 0.317 | 0.018 | 5.71 | 15 | 0.136 | 0.307 | 0.269 | 0.051 | 19.0 |
| 6400 | 15 | 0.171 | 0.198 | 0.185 | 0.009 | 4.9 | 15 | 0.093 | 0.184 | 0.155 | 0.029 | 18.8 |
| 12800 | 15 | 0.102 | 0.128 | 0.114 | 0.007 | 5.7 | 15 | 0.064 | 0.112 | 0.096 | 0.014 | 14.7 |
| 25600 | 15 | 0.046 | 0.086 | 0.073 | 0.009 | 11.9 | 15 | 0.052 | 0.084 | 0.069 | 0.009 | 13.2 |
| 51200 | 15 | 0.048 | 0.069 | 0.057 | 0.005 | 9.6 | 15 | 0.045 | 0.079 | 0.055 | 0.009 | 16.3 |
| 102400 | 15 | 0.043 | 0.057 | 0.049 | 0.004 | 8.3 | 15 | 0.036 | 0.068 | 0.044 | 0.008 | 18.6 |

*S3 Table. Intra Assay precision data*

| *Dilution Series* | *CN54 ELISA* | | | | | | *AIDSVAX ELISA* | | | | | |
| --- | --- | --- | --- | --- | --- | --- | --- | --- | --- | --- | --- | --- |
|  | Count | Min | Max | Average | STDEV | %CV | Count | Min | Max | Average | STDEV | %CV |
| 100 | 15 | 1.545 | 1.914 | 1.704 | 0.147 | 8.7 | 15 | 1.704 | 2.176 | 1.893 | 0.167 | 8.8 |
| 200 | 15 | 1.201 | 1.543 | 1.343 | 0.143 | 10.6 | 15 | 1.403 | 1.795 | 1.549 | 0.124 | 8.0 |
| 400 | 15 | 0.821 | 1.176 | 0.978 | 0.143 | 14.6 | 15 | 0.911 | 1.351 | 1.135 | 0.131 | 11.5 |
| 800 | 15 | 0.479 | 0.787 | 0.613 | 0.114 | 18.5 | 15 | 0.533 | 0.890 | 0.729 | 0.121 | 16.6 |
| 1600 | 15 | 0.288 | 0.472 | 0.366 | 0.069 | 18.9 | 15 | 0.269 | 0.545 | 0.435 | 0.074 | 17.1 |
| 3200 | 15 | 0.176 | 0.280 | 0.221 | 0.041 | 18.5 | 15 | 0.15 | 0.314 | 0.251 | 0.045 | 18.0 |
| 6400 | 15 | 0.103 | 0.163 | 0.132 | 0.021 | 16.2 | 15 | 0.12 | 0.180 | 0.153 | 0.018 | 11.7 |
| 12800 | 15 | 0.074 | 0.117 | 0.091 | 0.013 | 14.3 | 15 | 0.086 | 0.113 | 0.098 | 0.011 | 11.0 |
| 25600 | 15 | 0.059 | 0.073 | 0.065 | 0.005 | 7.6 | 15 | 0.06 | 0.089 | 0.071 | 0.009 | 12.3 |
| 51200 | 15 | 0.046 | 0.062 | 0.052 | 0.004 | 8.0 | 15 | 0.046 | 0.063 | 0.056 | 0.005 | 9.0 |
| 102400 | 15 | 0.042 | 0.052 | 0.046 | 0.003 | 7.3 | 15 | 0.042 | 0.062 | 0.050 | 0.005 | 10.8 |

*S4 Table. Inter Assay Precision data*

| *Dilution Series* | *CN54 ELISA* | | | | | | *AIDSVAX ELISA* | | | | | |
| --- | --- | --- | --- | --- | --- | --- | --- | --- | --- | --- | --- | --- |
|  | Count | Min | Max | Average | STDEV | %CV | Count | Min | Max | Average | STDEV | %CV |
| 100 | 15 | 1.574 | 2.030 | 1.777 | 0.151 | 8.% | 15 | 1.731 | 2.118 | 1.870 | 0.127 | 6.8 |
| 200 | 15 | 1.211 | 1.721 | 1.460 | 0.152 | 10.4 | 15 | 1.403 | 1.795 | 1.549 | 0.124 | 8.0 |
| 400 | 15 | 0.858 | 1.259 | 1.058 | 0.127 | 12.0 | 15 | 0.911 | 1.351 | 1.135 | 0.131 | 11.5 |
| 800 | 15 | 0.492 | 0.879 | 0.688 | 0.113 | 16.4 | 15 | 0.533 | 0.890 | 0.729 | 0.121 | 16.6 |
| 1600 | 15 | 0.292 | 0.533 | 0.410 | 0.074 | 18.0 | 15 | 0.269 | 0.545 | 0.435 | 0.074 | 17.1 |
| 3200 | 15 | 0.175 | 0.290 | 0.243 | 0.038 | 15.6 | 15 | 0.15 | 0.314 | 0.251 | 0.045 | 18.0 |
| 6400 | 15 | 0.113 | 0.174 | 0.148 | 0.021 | 14.0 | 15 | 0.12 | 0.180 | 0.153 | 0.018 | 11.7 |
| 12800 | 15 | 0.078 | 0.106 | 0.097 | 0.009 | 8.9 | 15 | 0.086 | 0.113 | 0.098 | 0.011 | 11.0 |
| 25600 | 15 | 0.059 | 0.091 | 0.071 | 0.008 | 11.4 | 15 | 0.06 | 0.089 | 0.071 | 0.009 | 12.3 |
| 51200 | 15 | 0.049 | 0.066 | 0.056 | 0.005 | 8.6 | 15 | 0.046 | 0.063 | 0.056 | 0.005 | 9.0 |
| 102400 | 15 | 0.044 | 0.063 | 0.051 | 0.006 | 11.4 | 15 | 0.042 | 0.062 | 0.050 | 0.005 | 10.8 |

*S5 Table. Inter Operator Precision (Intermediate) data*
